# Modification of gemcitabine with oxaliplatin in China for unresectable gallbladder cancer: a cost-effectiveness analysis

**DOI:** 10.3389/fpubh.2024.1432947

**Published:** 2024-12-13

**Authors:** Zhaoyan Chen, Fangyuan Tian

**Affiliations:** Department of Pharmacy, National Clinical Research Center for Geriatrics, West China Hospital, Sichuan University, Chengdu, Sichuan, China

**Keywords:** gallbladder cancer (GBC), gemcitabine, oxaliplatin, fluorouracil, cost effectiveness

## Abstract

**Background:**

The incidence of gall bladder cancer (GBC), one of the most prevalent bile duct malignancies, differs with ethnicity and geographic location. To treat unresected GBC in the Chinese setting, this study aimed to assess the financial effectiveness of a combination of modified gemcitabine and oxaliplatin.

**Methods:**

Data from a randomized controlled study in which individuals with metastatic GBC were treated with oxaliplatin and gemcitabine demonstrated improved survival. A Markov model is built to calculate the incremental cost–benefit ratio (ICER) from the viewpoint of Chinese society on the basis of clinical symptoms and disease development. One-way certainty and probability sensitivity analyses are used to describe the uncertainty in the model.

**Results:**

Compared with those of fluorouracil (FU) and folinic acid, the utility value of modified oxaliplatin combined with gemcitabine increased by 0.22QALY throughout the course of the 10-year simulation (FA). In a Chinese healthcare setting, the cost-effectiveness ratio (ICER) is $52765.59/QALY, with a 0% chance of cost–benefit at the WTP (willing-to-pay) level of $37697.00/QALY. The ICERs predicted by sensitivity analysis were not significantly affected by cost variations related to the management of Grade 3–4 AEs, the diagnostics used, or hospitalization expenditures.

**Conclusion:**

In a Chinese healthcare context, modified gemcitabine coupled with oxaliplatin (mGEMOX) is not a cost-effective treatment option for unresectable GBC.

## Introduction

Fewer than 5,000 new instances of gallbladder cancer (GBC) are detected in the United States each year, and rates vary by geographic region and race (1). For GBC, surgery is the sole treatment option. However, only a small number of patients are suitable for curative surgery, with the remainder receiving palliative care, due to the extent of the lesion (including locally advanced unresectable lesions due to local invasion of critical structures or lesions that metastasized beyond local regional boundaries) (2). Currently, there is no standard chemotherapy for treating GBC (3, 4). Gemcitabine and oxaliplatin have demonstrated effects on the biliary tract in patients with pancreatitis and GBC either alone or in combination with other treatments (5–7). The third-generation platinum drug oxaliplatin is substantially less nephrotoxic and emetic than large doses of cisplatin. A good substitute for gemcitabine and cisplatin may be modified gemcitabine coupled with oxaliplatin.

According to recent findings from a phase III single-center trial conducted in India, the combination of oxaliplatin and gemcitabine significantly prolonged median progression-free survival (PFS) (*p* < 0.001) and median overall survival (in months) (*p* = 0.039) in patients with unresectable GBC (8). Data on the top-selling pharmaceuticals worldwide indicate that oxaliplatin generated nearly $20 billion in sales over the 20-year period from 1999--2019. The cost of oxaliplatin has decreased somewhat in China due to escalating competition from generic medications. In Sichuan Province, a tube of oxaliplatin (50 mg) costs US$326.59. However, the cost-effectiveness of a pharmacological treatment plan is one of the factors that influences the ultimate selection in a nation such as China, which has inadequate medical resources. The potential cost advantages of the mGEMOX regimen for the treatment of patients with unresectable GBC were assessed in this study via a Markov model.

## Materials and methods

### Target population

Patients who were 18 years of age or older with unresectable GBC verified by biopsy or fine needle aspiration cytology met the inclusion criteria. If a patient has previously received adjuvant chemotherapy and/or radiotherapy, it should be completed at least 6 months before recruitment into this study. Everyone in the PFS health status group first received one of the two treatments. For a maximum of 6 cycles or until intolerable toxicity, whichever came first, the patients in the intervention group received 900 mg/m^2^ gemcitabine and 80 mg/m2 IV infusion (mGEMOX) oxaliplatin on days 1 and 8 of every 3 weeks. Patients in the comparison group received an intravenous bolus of FA 20 mg/m^2^ and FU 425 mg/m^2^ once a week for 30 weeks (FUFA).

### Model structure

**Figure 1:**
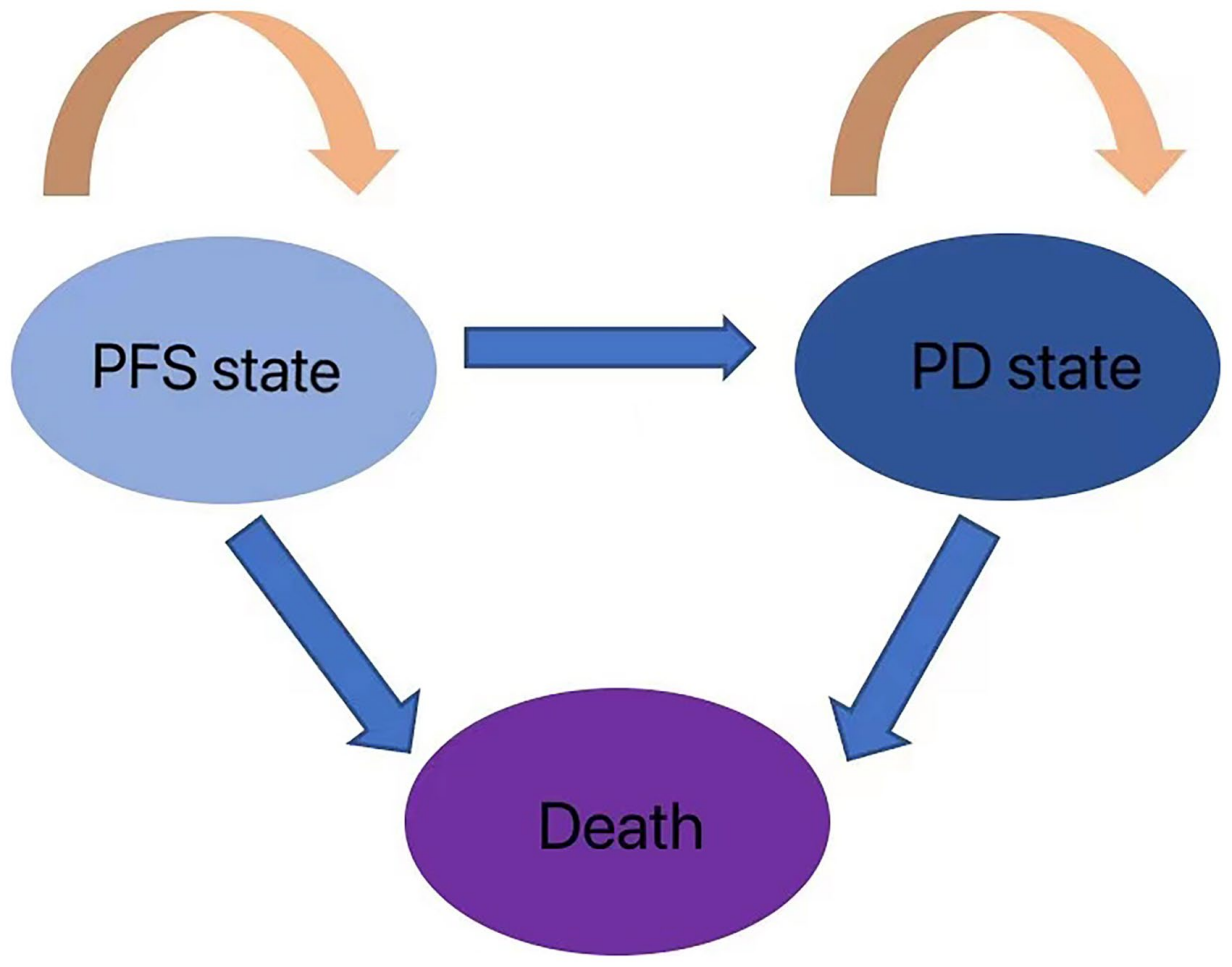
Markov model health states and transition rules.

The cost-effectiveness of the two treatment modalities was compared via the Markov decision tree model. Progression-free survival (PFS), progressive disease (PD), and death are the three mutually exclusive states included in the model. All patients with metastatic or unresectable GBC begin therapy for PFS; however, they may transition over time to other health states (Figure 1). Patients in the PFS state may enter PD or death states after a Markov cycle, or they may remain in the PFS state. Patients in the PD state, however, are unable to return to the PFS condition, and any patient may enter the death state. An absorbed state is the death state. Toxicology tests were performed on all patients. Toxicology was determined via the National Cancer Institute Common Toxicity Criteria (version 3.0). The model timeframe is set to 1 month on the basis of clinical symptoms and the rate of disease progression. The following formula was used to convert monthly transition probabilities from median survival estimates (Table 1): the formula *p* = 1-e-R, where R = -ln[0.5]/(time to incident/number of treatment cycles), was used to obtain P (1 month) = 1-(0.5, 1/median time to event), which was then used to calculate P (1 month) (9, 10). For this model, a 10-year time horizon was selected.

**Table 1:** Transition probabilities between unresectable GBC states.

Transition probabilities	Baseline value	Lower limit	Upper limit
mGEMOX			
P_pfs-pfs-1_	0.85	0.68	1.00
P_pfs-pd-1_	0.08	0.06	0.09
P_pfs-death-1_	0.07	0.06	0.08
P_pd-pd-1_	0.50	0.40	0.60
P_pd-death-1_	0.50	0.40	0.60
FUFA			
P_pfs-pfs-2_	0.68	0.54	0.82
P_pfs-pd-2_	0.18	0.14	0.22
P_pfs-death-2_	0.14	0.11	0.17
P_pd-pd-2_	0.53	0.43	0.64
P_pd-death-2_	0.47	0.37	0.56

FUFA, fluorouracil and folinic acid; mGEMOX, modified gemcitabine and oxaliplatin; PFS, progression-free survival; PD, progressive disease.

### Model parameters

Expenses were calculated with patient payments in mind (Table 2). Anticancer medications, diagnostics (total abdominal enhanced CT, biochemical examination), management of grade 3–4 adverse events (AEs), and hospitalization expenditures were taken into account during the analysis. Individual differences resulted in implicit costs being disregarded. We hypothesized that the average patient would weigh 65 kg, stand 1.64 m tall, and have a BSA of 1.72 m^2^ (11). The 2023 charge standards of West China Hospital, Sichuan University, were consulted to determine the unit price for each medication and test. We assessed the cost of second-line treatment in the two groups on the basis of the data from the trial by Sharma et al. (8) and the progression of the disease. The conversion rate used to convert all costs to US dollars was $1 = 6.82 (average exchange rate for January 2023) (12). This model uses health outcome data from single-center, randomized, controlled, open-label research. Years of quality-adjusted life are used to express the health utility value (QALY). Given that fundamental utility information is absent from the original literature, the health utility value is derived from published literature (13). The utility values for death status, progressive disease, and progression-free disease are 0.77, 0.64, and 0.00, respectively. Table 2 displays the model parameters for cost and effectiveness. The cost and utility value are both reduced at a 5% annual rate in accordance with the “China guidelines for pharmacoeconomic evaluations.”

**Table 2:** Model parameters related to cost and effectiveness in patients with GBC.

Parameters	mGEMOX	FUFA
Clinical efficacy, months		
Median PFS, months	8.5	3.5
Median OS, months	9.5	4.6
Probability of grades 3–4 adverse events, %		
Vomiting	7.69	7.14
Myelosuppression	38.46	7.14
Neurotoxicity	11.54	0
Transaminitis	15.38	0
Neutropenic fever	7.69	0
Unit costs, $/months		
Cost of tests	90.03	164.37
Hospitalization	140.76	164.22
Cost for adverse events	1.26	/
Cost for the progressive disease state	2017.94	1834.49
Annual discount rate, %	5	5

FUFA, fluorouracil and folinic acid; mGEMOX, modified gemcitabine and oxaliplatin; PFS, progression-free survival; PD, progressive disease.

### Sensitivity analyses

The findings of the deterministic one-way sensitivity analysis revealed variation in all the parameters utilized in the evaluation (with the exception of the discount rate; range = 20%). The discount percentage varied from 0 to 8%. A second-order Monte Carlo simulation was used to perform a probabilistic sensitivity analysis over the course of 1,000 iterations. To assess the most successful techniques at different willingness-to-pay (WTP) thresholds, cost-effectiveness acceptability analysis was performed. The WTP benchmark was set at three times China’s $37697.00/QALY GDP *per capita* in 2022.

## Results

### Base-case analysis

**Figure 2:**
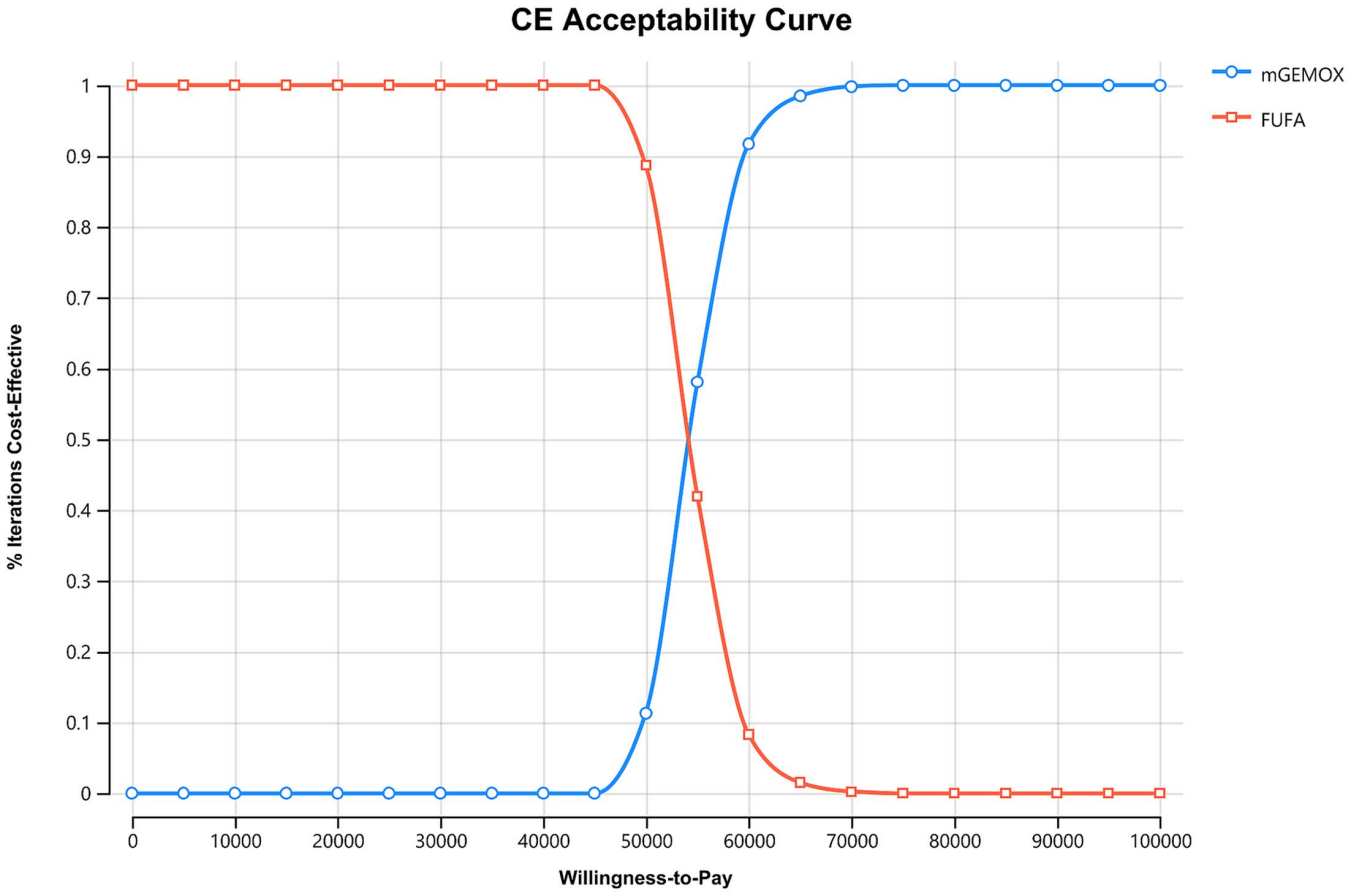
Cost-effectiveness acceptability curves. FUFA, fluorouracil and folinic acid; mGEMOX, modified gemcitabine and oxaliplatin.

The findings of the 10-year simulation demonstrate that the utility value of modified oxaliplatin in combination with gemcitabine is increased by 0.22QALY (0.45QALY vs. 0.23QALY) compared with that of FU and FA (Table 3). Similar to the cost increase, the cost-effectiveness ratio (ICER) shows a 0% chance of being cost-effective at the WTP level of $37697.00/QALY, and the cost increase is 11608.43 US dollars per person (Figure 2). Given its overall cost implications, this combination is not a cost-effective treatment option for unresectable gallbladder cancer. For patients in the PFS illness state, the cost of the mGEMOX regimen is more than 10 times greater than the cost of the FUFA regimen ($12790.39 vs. $1121.48).

**Table 3:** The results of the cost-effectiveness analysis.

Parameters	mGEMOX	FUFA
Costs for the PFS state, $	12790.39	1121.48
Costs for the PD state, $	2111.92	2172.40
Total costs, $	14902.31	3293.88
Incremental costs, $	11608.43	/
Effectiveness for the PFS state, QALYs	0.39	0.17
Effectiveness for the PD state, QALYs	0.06	0.06
Total effectiveness, QALYs	0.45	0.23
Incremental effectiveness, QALYs	0.22	/
Total C/E, $/QALY	33116.24	14321.22
ICER, $/QALY	52765.59	/

### Sensitivity analysis

**Figure 3:**
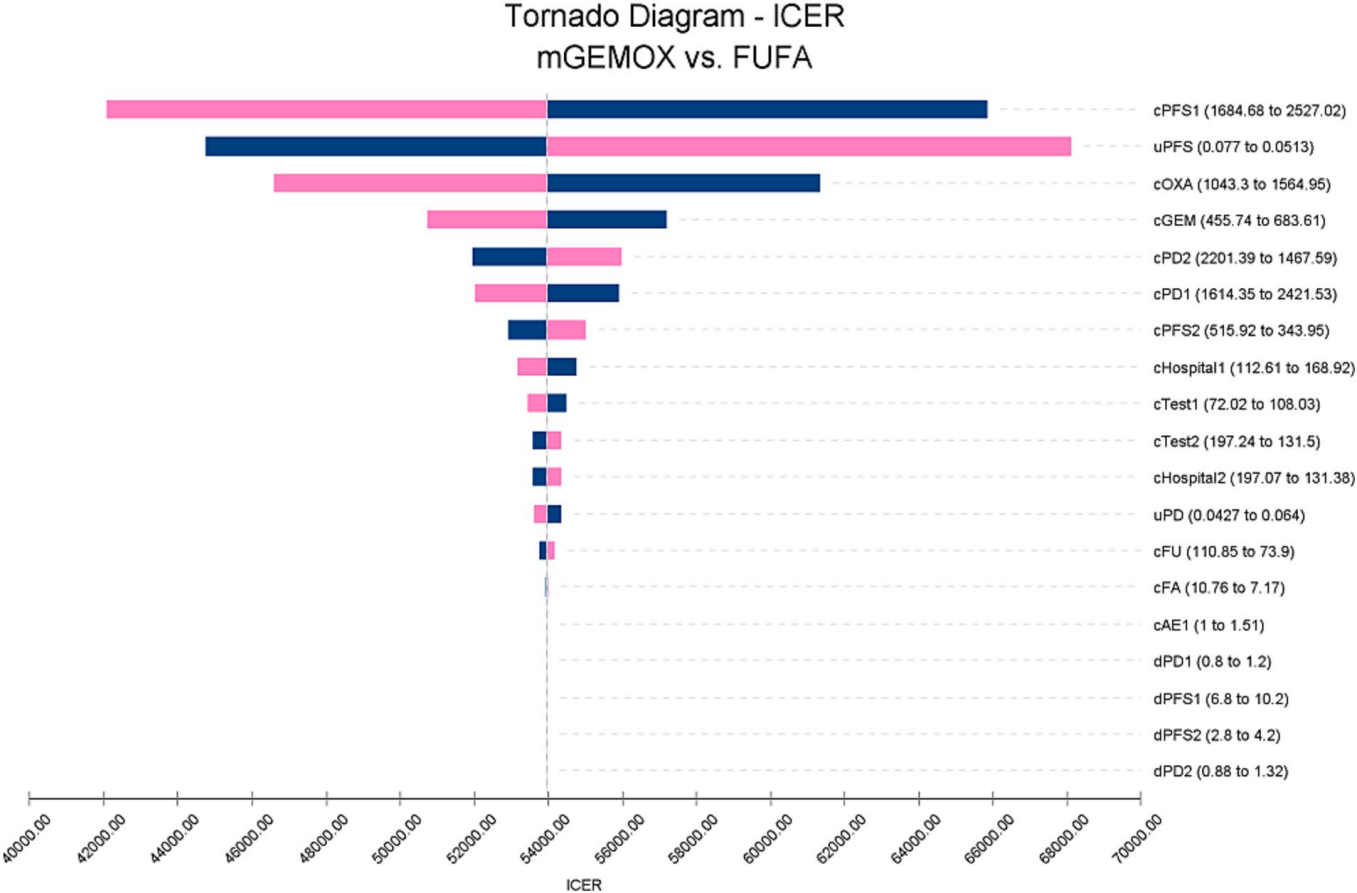
Tornado diagram of one-way sensitivity analysis. c, Costs of a specific group; u, Utilities of a specific group; d, Duration of a group; 1, mGEMOX group. 2, FUFA group.

**Figure 4:**
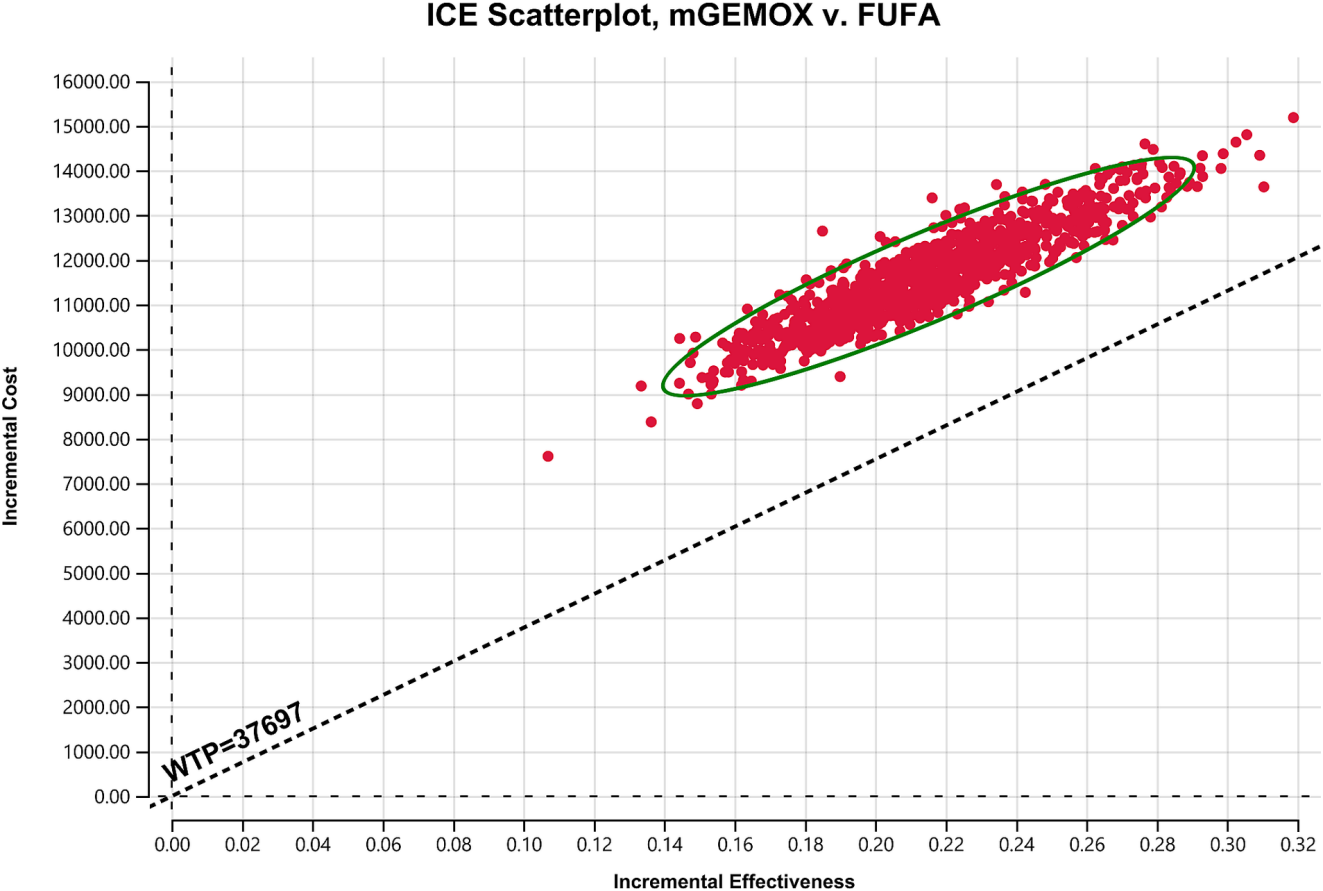
Scatter plot of the probabilistic sensitivity analysis results. FUFA, fluorouracil and folinic acid; mGEMOX, modified gemcitabine and oxaliplatin.

To evaluate the effects of specific Markov model parameters, a one-way sensitivity analysis was performed. An illustration of the outcomes is shown in a tornado diagram (Figure 3). The most important model parameters were the cost of oxaliplatin for the mGEMOX group, the utility of the PFS state, and the cost of the PFS state, all of which exhibited a variance of approximately 20%. The ICER increased from $42068.81/QALY to $65850.30/QALY as the PFS state cost changed from $1684.68/month to $2527.02/month. The usefulness of PFS increased from 0.62 to 1.00, which resulted in a decrease in the ICER from $68112.01/QALY to $41417.29/QALY. The ICER values anticipated by sensitivity analysis were, however, less affected by changes in the expenses associated with managing grade 3–4 adverse events (AEs), the tests used, or the hospital fees spent. The ICER was also consistently higher than $37697.00/QALY, as shown by probabilistic sensitivity analysis (1,000 iterations) (Figure 4).

## Discussion

This study is the first to examine the cost-effectiveness of modified oxaliplatin and gemcitabine regimens with FU and FA in Chinese patients with unresectable GBC on the basis of findings from a literature search. Adopting the viewpoint of the healthcare system in China. We used the most recent price for first-class hospitals in operation.

To calculate the ICER, we selected the Markov model. Given that PFS and OS are longer in the mGEMOX group, which is consistent with clinical trial data, the overall utility of the mGEMOX group is greater (8). Chinese patients typically have greater utility values than do patients in some other industrialized nations because they frequently conceal their genuine ailments, and doctors primarily notify their families (14). China is a vast country with uneven development across different regions. In areas with stronger traditional beliefs, patients tend to rely more on the advice of family members and may conceal some of their symptoms. This contrasts sharply with the more individualistic approach to seeking medical treatment commonly seen in major cities (15). Moreover, China’s medical resource allocation also suffers from significant imbalances, primarily manifested in notable disparities between urban and rural areas, among different regions, and across various tiers of medical institutions. This imbalance results in inequalities in the quality and accessibility of medical services, which have profound impacts on residents’ health. Policy adjustments and resource optimization are necessary to narrow these gaps and enhance the overall level of medical care (16). This viewpoint is also reflected in the sensitivity analysis. We discovered that although the cost acceptance rate of the mGEMOX group steadily increases when the willingness-to-pay threshold for each QALY is between US$37697.00 and US$52765.59, it is still not cost-effective. The test composition is a cost-effective option only when the WTP value is more than $52765.59/QALY compared to the control group.

The third-generation platinum anticancer drug is oxaliplatin. It is a platinum-based diaminocyclohexane chemical that is effective against ovarian and colorectal cancer. It was introduced in France in 1996, and the FDA gave its approval in 2002. A new cytosine nucleoside derivative called gemcitabine works mostly in the G1/S phase. The NCCN advised gemcitabine and oxaliplatin for biliary cancers because of their survival advantage (17), but there are variations in the regimens’ economic reports among nations. In Japan, treating advanced biliary tract cancer with cisplatin, gemcitabine, and gemcitabine is not cost effective (18). A cost-effective therapeutic option for advanced biliary cancer in the US is cisplatin with gemcitabine, which can replace gemcitabine as a single drug (13). By examining the treatment of advanced biliary tract cancer from the standpoint of China’s health service system, Chen et al. (19) demonstrated that the capecitabine + oxaliplatin regimen is more cost effective than the gemcitabine + oxaliplatin regimen as a first-line therapy. However, our data demonstrate that the modified gemcitabine + oxaliplatin strategy is not more affordable than the fluorouracil + calcium combination (19). The median PFS for the mGEMOX group was reportedly 8.5 months, whereas it was 3.5 months for the FUFA group. Compared with those of fluorouracil and leucovorin, the utility value of modified oxaliplatin combined with gemcitabine improved by 0.22 QALYs after the model was run for 10 years, but only at the current Chinese exchange rate ($37697.00/QALY). Owing to the increase in overall cost, modified gemcitabine plus oxaliplatin is not an affordable treatment option for unresectable GBC. The cost ratio between the two patient groups with PFS disease status reached a high of 11.4%.

The guidelines suggest the use of oxaliplatin, a member of the third-generation platinum class, as the initial therapy for a number of tumor types. Another representative Phase II study also demonstrated the clinical efficacy of this regimen. This study included 31 patients with previously untreated advanced biliary cancer (19 of whom had gallbladder cancer), all with good performance status and serum bilirubin levels below 2.5 times the upper limit of normal (ULN). When treated with gemcitabine (1,000 mg/m^2^, administered on Day 1) + oxaliplatin (100 mg/m^2^, administered on Day 2) every 2 weeks, the response rate was 36% and the median overall survival was 14.3 months (20). The cost of medical insurance in China has dramatically decreased in recent years as a result of numerous discussions, although the ICER is still significantly greater than the WTP level. Patients with unresectable GBC now have a chance of survival due to mGEMOX; however, the different treatment methods used in the Chinese medical system are financially hindered by high drug prices and a lack of medical resources. Provinces with high GDP, however, should consider adding oxaliplatin paired with gemcitabine to the local supplemental list, given the encouraging treatment gains reported.

In this study, the Markov decision tree model was utilized to simulate disease progression. However, certain limitations should be noted: the extrapolation is inadequate, and the cost–benefit analysis relies on clinical trial data rather than real-world studies. For the studied population, medical costs were sourced from the Sichuan Province Drug Price Publicity Network and adjacent hospitals. Additionally, the trial data employed in this research originated from local hospitals in India, with no Chinese patients participating in the trials. Consequently, the findings are more suitable for guiding health policy decisions in western China.

In conclusion, the findings of this study demonstrate that, from the perspective of Chinese society, mGEMOX is not economically advantageous for GBC patients compared with FUFA. To make mGEMOX more relevant for this patient population, it should be considered to appropriately reduce costs and offer social aid.

## Data Availability

The original contributions presented in the study are included in the article/supplementary material, further inquiries can be directed to the corresponding author.
